# European Medicinal Leeches—New Roles in Modern Medicine

**DOI:** 10.3390/biomedicines8050099

**Published:** 2020-04-27

**Authors:** Sarah Lemke, Andreas Vilcinskas

**Affiliations:** 1 Institute for Insect Biotechnology, Justus-Liebig-University Giessen, Heinrich-Buff-Ring 26-32, D-35392 Giessen, Germany; Sarah.Lemke@agrar.uni-giessen.de; 2 Fraunhofer Institute for Molecular Biology and Applied Ecology IME, Department of Bioresources, Ohlebergsweg 12, D-35392 Giessen, Germany

**Keywords:** medicinal leeches, drug discovery, *Hirudo* spec., antistasins, hirudin, eglins, saratins

## Abstract

Before the advent of modern medicine, natural resources were widely used by indigenous populations for the prevention and treatment of diseases. The associated knowledge, collectively described as folk medicine or traditional medicine, was largely based on trial-and-error testing of plant extracts (herbal remedies) and the use of invertebrates, particularly medicinal maggots of the blowfly *Lucilia sericata* and blood-sucking leeches. The widespread use of traditional medicine in the West declined as scientific advances allowed reproducible testing under controlled conditions and gave rise to the modern fields of biomedical research and pharmacology. However, many drugs are still derived from natural resources, and interest in traditional medicine has been renewed by the ability of researchers to investigate the medical potential of diverse species by high-throughput screening. Likewise, researchers are starting to look again at the benefits of maggot and leech therapy, based on the hypothesis that the use of such animals in traditional medicine is likely to reflect the presence of specific bioactive molecules that can be developed as drug leads. In this review, we consider the modern medical benefits of European medicinal leeches based on the systematic screening of their salivary proteins.

## 1. The Biology of Medicinal Leeches

European medicinal leeches of the genus *Hirudo* are blood-feeding annelids. The most relevant species are *H. orientalis* (Asian leech), *H. medicinalis* (European leech) and *H. verbana* (Hungarian leech). All three species are ectoparasites that live in freshwater ponds and slowly flowing streams, where they locate their vertebrate hosts by sensing heat, chemicals or movement [1,2]. Leeches attach to the host body surface and cut the skin using hundreds of calcified teeth [3]. They can then draw blood for up to one hour while secreting saliva into the wound. The secreted salivary proteins and peptides reach the vascular system of the host via thousands of tiny salivary gland cell ducts [4]. After ingestion by the leech, the host blood is compressed in the crop by the excretion of water and salts [5,6]. The remaining highly viscous blood comprises plasma proteins and blood cells and can be stored in the crop for up to one year [7]. It is thought that the morphology of the concentrated erythrocytes remains stable during storage [8], which means that proteolysis induced by host proteases released from leukocytes is inhibited [5]. Furthermore, leeches inevitably make contact with (and thus ingest) some bacteria on the surface of the host’s skin during feeding, but the stored blood does not become overrun with pathogens. Indeed, foremost symbiotic core bacteria such as *Aeromonas veronii*, *A. hydrophila* and *Rikinella*-like species survive in the alimentary tract of the leech [9,10,11]. It is supposed that symbionts like *A. veronii* support the digestion of host blood by facilitating hemolysis [10,12,13] and may also help to suppress the growth of other bacteria in the crop of the leech [9]. In most parasitic leeches the host blood is stored in the crop, while food digestion and the absorption of nutrients occur predominantly in the intestine. It can be assumed that medicinal leech enzymes (e.g., endopeptidases, aminopeptidases, phosphatases) promote digestion processes [14].

## 2. The Pharmacological Potential of Medicinal Leeches

Medicinal leeches were used by Egyptian, Indian, Greek and Arab physicians thousands of years ago. The main application was bloodletting, but leeches were also recommended for the treatment of systemic ailments such as inflammation, skin diseases, rheumatic pain or problems with the reproductive system [15]. As an advocate of leech therapy, the Greek physician Galen of Pergamon (130–201 AD) described leeches as an effective treatment for numerous diseases. Later, in the Middle Ages, leech therapy was popular because it was less painful than conventional treatments and was recommended even for diseases of the nervous system and eyes. The use of leeches declined in the age of modern medicine, but medical interest was rekindled when one of the strongest natural anticoagulants—hirudin—was discovered in leech saliva by John Berry Haycraft in 1884, further characterized by Fritz Markwardt in the 1950s [15].

In the 1960s, physicians rediscovered the pharmacological potential of leech saliva. For example, medicinal leeches were used to prevent vascular disorders after reconstructive surgery [16], to re-establish disrupted blood vessel networks and as an alternative to anti-inflammatory drugs. Most reports concerning medicinal leech therapy focus on cosmetic and reconstructive surgery. However, leech therapy has been tested for many conditions over the past two decades, including migraine [17,18], knee osteoarthritis [19,20,21,22,23,24,25], cardiovascular disease [26,27,28], skin disorders [29], diabetic foot ulcers [30,31,32], priapism [33], macroglossia [34,35], cancer [36,37] and skin wounds [38,39]. For most of these conditions only individual case studies were published [39], but migraine and knee osteoarthritis are exceptions. Migraine is a primary neurological disorder and, for most patients, a lifelong illness associated with headaches, vomiting, nausea, photophobia and phonophobia. In a case series of seven patients who were unresponsive to conventional drugs, post-auricular leech therapy was shown to significantly reduce the frequency of migraine headaches, which the authors attributed to the presence of potent anesthetic, anti-inflammatory and vasodilator substances in the leech saliva [17]. Osteoarthritis is a disorder of the joints that is prevalent in older people (>65 years) and causes pain after activity and stiffness after rest [40]. A meta-analysis of seven articles published between 2000 and 2017 showed that leech therapy could improve the symptoms of knee osteoarthritis and reduce pain [39]. Importantly, leeches placed on the knee often achieved comparable or even better pain relief than conventional drugs, and patients reported that mobility was restored and the benefits of leech therapy were sometimes still evident after six months [41]. Although the benefits of leech therapy were evident from these studies, the salivary compounds responsible for these effects and the underlying molecular mechanisms were not characterized in detail.

## 3. Salivary Proteins: Natural Drugs from Medicinal Leeches

Antagonistic interactions between parasites and their hosts have led to an evolutionary “arms race”, during which ectoparasites adapted to feed on host body fluids [42,43]. To ingest and digest host blood, medicinal leeches synthesize more than 100 salivary proteins and peptides [44,45,46,47]. The molecules are secreted during feeding and target physiological pathways involved in host defense, working as analgesics (kininases), anticoagulants (hirudin, calin, saratin and apyrase), anti-inflammatories (eglins, bdellins and tryptase inhibitor), cell matrix-degrading proteins (hyaluronidase) or antimicrobials [7,45,47,48,49,50,51,52,53,54,55,56,57,58]. Salivary transcriptome data from *Macrobdella decora* [59] and *Hirudo nipponia* [60], as well as expressed sequence tag libraries constructed from the salivary glands of *H. verbana*, *M. decora* and *Aliolimnatis fenestrata* [61], provided insight into the spectrum proteins found in leech saliva. For European medicinal leeches, the combined transcriptomic analysis of salivary gland cells and proteomic analysis of saliva in *H. medicinalis*, *H. orientalis* and *H. verbana* revealed a much wider repertoire of components than previously known [44], indicating that only ~15% of the salivary proteins in these species were identified and characterized (Table 1).

Analysis of the salivary transcriptomes of *H. medicinalis*, *H. orientalis* and *H. verbana* revealed the presence of transcripts representing 189, 86 and 344 salivary proteins, respectively [44]. The three closely related species were found to share 39 orthologous clusters, whereas 50 orthologous clusters were shared by any two of the three species [44]. Many of these newly discovered leech salivary proteins are either associated with blood feeding or related to proteins found in animal venoms [44]. The salivary proteins predicted from transcriptomic and proteomic data can be assigned to various functional groups based on their structural similarities, including metalloproteases representing the M12, M13 and M28 families, hyaluronidases, apyrases, adenosine deaminases, antistasins, cysteine-rich secretory proteins (CRISPs), eglins, cystatins, PAN/apple domain proteins, α2-macroglobulins, low-density lipoprotein receptors, R-type lectins, and salivary proteins containing a von Willebrand factor type A (vWA) domain. These proteins are likely to be involved in the regulation of blood coagulation, the temporary adjustment of blood pressure, the regulation of inflammation, the suppression of microbial growth or the digestion of blood in the crop [44]. Interestingly, differential gene expression analysis indicated that genes encoding salivary proteins, such as hirudin, eglins, saratins and destabilases, were also expressed in other leech tissues, showing that at least some leech “salivary proteins” are not restricted to the saliva and may have additional physiological functions [44]. Some leech-specific anticoagulants were also found in leeches that do not feed on blood, such as *Whitmania pigra* [62]. Interestingly, these anticoagulants were upregulated after feeding [62] just as they are in blood-feeding leeches [63].

The identified metalloprotease families in leech salivary encompass astacins (M12), neprilysins (M13) and aminopeptidase S (M28). Members of these metalloprotease families were also determined in the salivary secretion of medicinal maggots of *Lucilia sericata* [64]. Astacin-like metalloproteases are endopeptidases, which were originally identified in the crayfish *Astacus astacus*, which contribute to digestion. A homologues were found in the venom of the brown spiders *Loxosceles,* with the recombinant form able to induce morphological changes, such as loss of adhesion of muscular aorta cells in vitro and hydrolyzed purified fibrinogen and fibronectin [65]. Mammalian neprilysin is involved in reproduction and the modulation of neuronal activity and blood pressure [66]. Interestingly, the transcriptomic analysis of the salivary glands from medicinal maggots *L. sericata* elucidated a diversification of proteolytic enzymes [64], whereas the most diverse groups of molecules in the saliva of leeches represented protease inhibitors.

**Table 1:** Leech salivary proteins from *H. medicinalis, H. verbana* or *H. orientalis.* Isoforms of individual proteins are not shown.

Salivary Protein	Mechanism of Action	Biological Significance	Reference
Hirustasin (Mass: 5.866 kDa)	Tissue kallikrein inhibitor and inhibitor of trypsin, chymotrypsin and neutrophil cathepsin G	Anti-inflammatory	[55]
Apyrase (Mass: 45 kDa)	Cleavage of adenosine 5′-diphosphate	Inhibitor of platelet aggregation	[67]
Bdellin B-3 (Mass: 6.141 kDa)	Inhibitor of plasmin, trypsin and sperm acrosin	Anti-inflammatory	[68]
Calin (Mass: 65 kDa)	Prevents the binding of von Willebrand factor to collagen	Inhibitor of platelet aggregation	[50,69]
Collagenase (Mass: 50 kDa)	Cleavage of collagen	Collagen digestion	[70]
Destabilase (Mass: 12.6–12.9 kDa)	Cleavage of fibrin clots, cleavage of peptidoglycans in bacterial walls	Anticoagulant/antimicrobial	[71,72,73]
Eglin C (Mass: 8.1 kDa)	Neutrophil elastase inhibitor, cathepsin G inhibitor	Anti-inflammatory	[74,75]
Hirudin (Mass: 7.1 kDa)	Thrombin inhibitor	Anticoagulant	[48,76]
Hirudin-like factors (Mass: 4.27–6.67; isoforms HLF1-HLF3)	Unknown for the three European species		[77,78]
Hyaluronidase (Mass: 27.5 kDa)	Cleavage of hyaluronic acid	Extracellular matrix digestion	[79]
Leech-derived tryptase inhibitor (Mass: 4.7 kDa)	Mast cell tryptase inhibitor	Anti-inflammatory	[56,57]
Leech carboxypeptidase inhibitor (Mass: 7.2 kDa)	Carboxypeptidase B inhibitor	Unclear	[80]
Saratin (Mass: 12 kDa)	Inhibits the binding of von Willebrand factor to collagen	Inhibitor of platelet aggregation	[51,81]
Yagin (Mass: 15.4 kDa)	Factor Xa inhibitor	Anticoagulant	[82]

Many leech salivary proteins, including antistasin-like inhibitors, hirudins, hirudin-like factors and Kunitz-type proteinase inhibitors, show remarkable diversity [44,78], possibly reflecting target-oriented evolution [83] promoted by gene duplication events [84]. Gene duplication events are likely to have promoted the acquisition of two major salivary protein families—salivary blood coagulation inhibitors and platelet aggregation inhibitors—in blood-feeding ticks [85]. Gene recruitment also supports the diversification of salivary protein isoforms, based on the hypothesis that regulatory evolution is fundamental for adaptive evolution [86]. Accordingly, at least some venom and salivary proteins were recruited from other tissues, where they fulfilled distinct biological functions. The recruitment of alternative splice variants and 5′ exon evolution might explain the adaptation of vampire bats to hematophagy and may be a more common source of genomic complexity in sanguivorous animals than the evolution of new genes [86]. This led to the identification of novel and convergently recruited venom proteins in blood-feeding leeches and vampire bats [86].

Evolutionary models explaining the adaptation of leech salivary proteins to specific hosts are still a matter of debate. Current challenges include the lack of well-characterized proteins in terms of mode of action and target. The isoproteins in leech saliva may have more than one target in the host, or their activity may be dependent on pH, temperature, the season or the developmental phase. Both the redundancy of salivary proteins (multiple proteins directed against the same target) and the potential cooperative interactions among multiple salivary proteins should be considered. The interplay of several salivary proteins can be seen in the bloodsucking arthropod *Rhodnius prolixus*, which produces four isoforms of salivary nitrophorin. All of them are vasodilators (working in cooperation) and histamine suppressors, but one is a strong inhibitor of factor IXa, another is a weaker anticoagulant and the remaining two isoforms appear to have lost their anticoagulant activity [87].

## 4. Antistasins as a Representative Leech Salivary Protein Family

Several antistasins were identified in leech species, including (1) the prototype antistasin, isolated from the salivary glands of the Mexican leech, *Haementeria officials*; (2) hirustasin and bdellastasin, identified in *H. medicinalis*; (3) ghilanten, identified in *Haementeria ghilianii*; (4) piguamerin, identified in *H. nipponia*; and (5) guamerin I (*H. nipponia*) and guamerin II, isolated from *Whitmania edentula* [88,89,90,91,92,93]. All of these cysteine-rich proteins contain several repeated motifs, each consisting of six conserved cysteine and two conserved glycine residues [94], but differ widely in terms of structure and function [89].

The prototype antistasin is a polypeptide of 119 amino acids that includes 10 disulfide bridges and a twofold internal repeat, suggesting that it arose following a gene duplication event [54,95]. This protein is a potent competitive inhibitor of coagulation factor Xa, a serine protease which cleaves antistasin at position Arg^34^ to yield a 10-kDa fragment [91]. The presence of antistasin therefore maintains host blood in a liquid state [54]. The medical applications of antistasin are not restricted to its role as an anticoagulant because its ability to inhibit serine proteases was also shown to prevent the spread of tumors, probably by reducing the likelihood of metastasis [96].

Additional antistasin-type proteins known to inhibit factor Xa include ghilanten [93] and yagin [82]. In contrast, guamerin I [97] and guamerin II [88] are specific inhibitors of neutrophil and pancreas elastases, whereas hirustasin is a potent inhibitor of trypsin, chymotrypsin, cathepsin G and tissue kallikrein [55]. In contrast to hirustasin, piguamerin does not inhibit tissue kallikrein, but does inhibit plasma kallikrein and trypsin [89]. The P1 residue of the reactive site determines the specificity of serine protease inhibitors [98]. If it is lysine or arginine, the inhibitor targets trypsin and trypsin-like enzymes. However, if it is tyrosine, phenylalanine, leucine or methionine, then chymotrypsin or chymotrypsin-like enzymes are more likely targets [99]. If it is alanine or serine, the inhibitor will tend to target elastase-like enzymes [98,99]. This was confirmed for a serine protease inhibitor containing antistasin and whey acidic protein (WAP) domains (StmAW-SPI) isolated from the tropical sea cucumber *Stichopus monotuberculatus* [98].

## 5. Leech Salivary Proteins as Drug Leads

**Figure 1:**
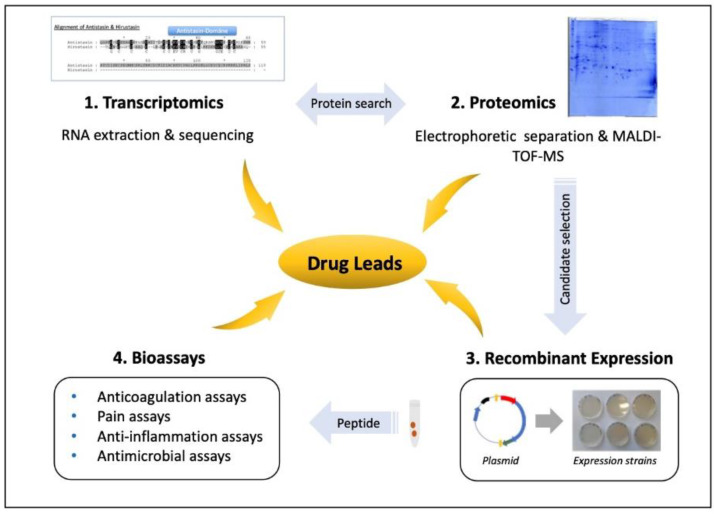
Workflow for the analysis of leech salivary proteins as drug leads. To analyze individual leech salivary proteins, a combination of transcriptomics and proteomics provides the protein sequences. Recombinant proteins are expressed to test their activities in cells, tissues and animal models, for example, to determine whether they possess anticoagulation, analgesic, anti-inflammatory or antimicrobial effects.

Natural products from plants and animals provide an astonishingly diverse source of active compounds for drug development and clinical trials [100] and can be used as tools for pharmacological or biotechnological applications [101]. Medicinal leeches are promising for the treatment of diseases associated with pain, inflammation or blood disorders. However, the use of living animals poses a risk of infection. Leeches carry bacteria in their digestive tract [10,11] and on their skin, and these bacteria could infect patients undergoing treatment. The use of antibiotic prophylaxis to minimize post-operative leech-borne infections only partially addresses this issue and encourages the emergence of multidrug-resistant pathogens in a clinical setting [102]. One strategy to avoid contact with leeches altogether is the extraction and purification of individual salivary components and their production as recombinant proteins to be administered using sterile equipment. Linked sets of proteomic and transcriptomic data are needed to explore bioactive proteins and peptides derived from natural animal sources such as leech saliva [101]. Such combined analysis (e.g., RNA-Seq + MALDI-TOF-MS or NanoLC-ESI-MS) allows researchers to compare salivary gland transcripts containing signal peptides with salivary proteins secreted into the host wound. Because European medical leeches have thousands of single salivary glands cells and their saliva secretion mechanisms are still unknown, it is necessary to prepare salivary gland cell tissues for proteomics. Comparative proteomic analysis of unfed leeches and fed leeches enabled researchers to separate proteins and to distinguish between secretory and nonsecretory proteins [63]. The combination of proteomics and transcriptomics followed by a conserved domain search made it possible to predict the functional domains of salivary proteins that may be responsible for the observed therapeutic effects of leeches, leading to the identification of new anti-inflammatory, analgesic or pro-coagulant leads (Figure 1). The pharmacological potential of a protein can only be established if its target is known, and this is best achieved by expressing the drug lead as a recombinant protein so that ample amounts are available for testing in vitro, in cells, in tissue-based assays and in animal models. Multiple assays are available for the detection of targets related to blood coagulation, pain pathways, antimicrobial activity, cytotoxicity and inflammation. Recombinant leech proteins can be expressed in bacteria [77,103], yeast [104,105,106], insect cells [107] or a cell-free expression system, or leech peptides can be prepared by chemical synthesis. Correct folding is important but difficult to control, because leech salivary proteins often contain numerous cysteine residues that form disulfide bonds and these structures must be replicated to ensure that synthetic and recombinant proteins remain stable and functional. Eglins are an identified leech salivary protein family without cysteine residues [74,75], while other described salivary proteins possess six cysteine residues (hirudin, hirudin-like factors, leech-derived tryptase inhibitor, bdellin-B3; saratin; [48,51,56,57,68,76,77,81]), eight cysteine residues (leech carboxypeptidase inhibitor; [80]), 10 cysteine residues (hirustasin; [55]) or 14 cysteine residues (destabilase; [71,72,73]). The formation of disulfide bonds is one of the most important post-translational modifications, ensuring the bioactivity of the protein and underpinnig the assignment of the protein to a given class or family [101]. Correct folding can be confirmed by X-ray crystallography or nuclear magnetic resonance (NMR) spectroscopy, but large quantities of protein are required. In contrast, preliminary structural analysis with limited sample quantities is possible using approaches such as electron capture dissociation (ECD) or electron transfer dissociation (ETD) coupled with liquid chromatography mass spectrometry (LC-MS) using a triple quadrupole ion trap mass spectrometer [108].
